# Supplementary material for: The interactome of intact mitochondria by cross-linking mass spectrometry provides evidence for coexisting respiratory supercomplexes
Source: Mol Cell Proteomics. 2017 Dec 8;17(2):216–32. doi: 10.1074/mcp.RA117.000470 (PMC5795388; doi:10.1074/mcp.RA117.000470)
Supplement: Supplemental Data [file supp_RA117.000470_133922_0_supp_23978_fzffwf.zip › spectra_annotation/mito_spectra_annotation/434-1-6-1-11-1.pdf]

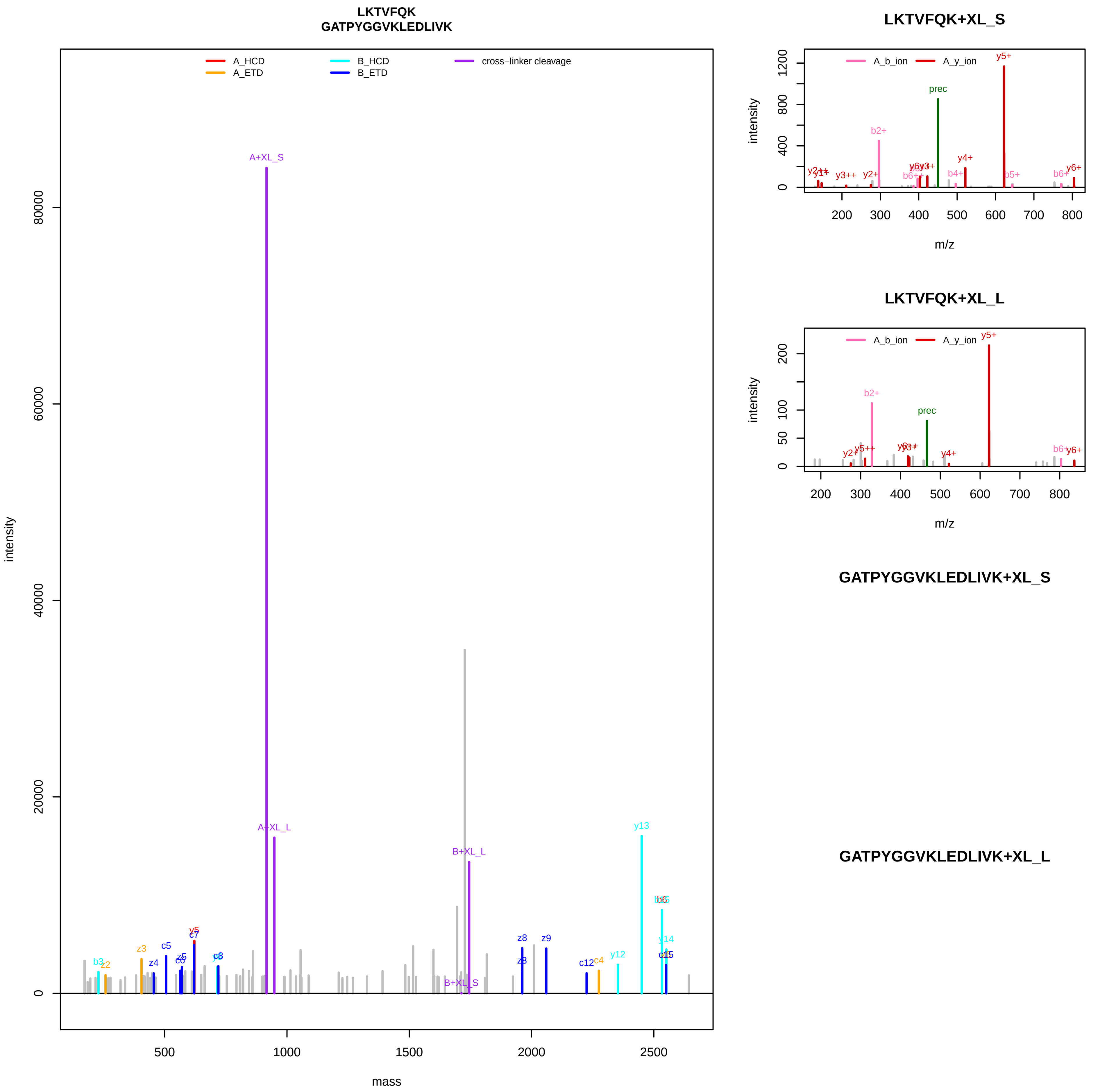
LKTVFQK
GATPYGGVKLEDLIVK
A_HCD
A_ETD
B_HCD
B_ETD
cross−linker cleavage
A+XL_S
A+XL_L
B+XL_L
B+XL_S
y13
y12
y14
c15
c12
c4
z8
z9
z3
c5
c6
c7
c8
y5
z3
z4
z2
z5
b3
intensity
mass
0
20000
40000
60000
80000
500
1000
1500
2000
2500
LKTVFQK+XL_S
A_b_ion
A_y_ion
prec
b2+
y5+
y4+
y6+
b6+
b4+
b5+
y3++
y2+
m/z
LKTVFQK+XL_L
A_b_ion
A_y_ion
prec
b2+
y5+
y4+
y6+
b6+
y5++
y2+
m/z
GATPYGGVKLEDLIVK+XL_S
GATPYGGVKLEDLIVK+XL_L
